# *N*-Methyl-*N*-(2-methyl­phen­yl)acetamide

**DOI:** 10.1107/S1600536810022361

**Published:** 2010-06-16

**Authors:** Zhang-Wei Yan, Yu-Hao Li, Qi Xiao, Liang Zhao, Hong-Jun Zhu

**Affiliations:** a Department of Applied Chemistry, College of Science, Nanjing University of Technology, Nanjing 210009, People’s Republic of China

## Abstract

In the title compound, C_10_H_13_NO, the N atom and the methyl group are almost coplanar with the benzene ring to which they are bonded [deviations of 0.131 (1) and 0.038 (1) Å, respectively, from the ring plane]. In the crystal structure, inter­molecular C—H⋯O hydrogen bonds form a three-dimensional network. Mol­ecules are stacked parallel to the *b*-axis direction.

## Related literature

For the use of related compounds as inter­mediates in syntheses of ligands for human β-amyloid plaques and for the preparation of the title compound, see Cai *et al.* (2007 ▶). For the use of related compounds in *N*-substituted glycine peptoid oligomers, see Shah *et al.* (2008 ▶). For a related structure, see: Li *et al.* (2008 ▶). For bond-length data, see: Allen *et al.* (1987 ▶)
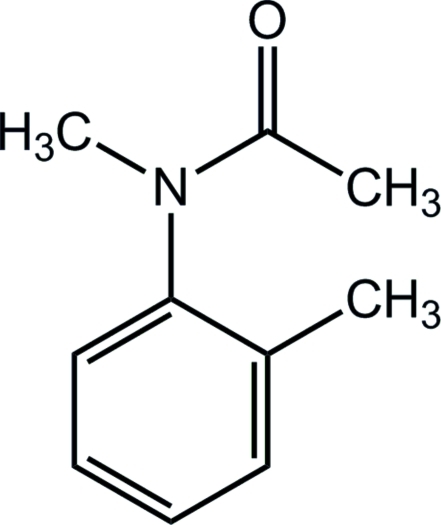


## Experimental

### 

#### Crystal data

C_10_H_13_NO
*M* *_r_* = 163.21
Monoclinic, 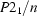

*a* = 11.288 (2) Å
*b* = 6.900 (1) Å
*c* = 12.234 (2) Å
β = 94.88 (3)°
*V* = 949.5 (3) Å^3^
*Z* = 4
Mo *K*α radiation
μ = 0.07 mm^−1^
*T* = 293 K
0.30 × 0.20 × 0.10 mm

#### Data collection

Enraf–Nonius CAD-4 diffractometer
Absorption correction: ψ scan (North *et al.*, 1968 ▶) *T*_min_ = 0.978, *T*_max_ = 0.993
3465 measured reflections
1726 independent reflections
1044 reflections with *I* > 2σ(*I*)
*R*_int_ = 0.055
3 standard reflections every 200 reflections  intensity decay: 1%

#### Refinement

*R*[*F* ^2^ > 2σ(*F* ^2^)] = 0.070
*wR*(*F* ^2^) = 0.180
*S* = 1.00
1726 reflections
112 parameters
4 restraints
H-atom parameters constrained
Δρ_max_ = 0.32 e Å^−3^
Δρ_min_ = −0.16 e Å^−3^

### 

Data collection: *CAD-4 Software* (Enraf–Nonius, 1985 ▶); cell refinement: *CAD-4 Software*; data reduction: *XCAD4* (Harms & Wocadlo,1995 ▶); program(s) used to solve structure: *SHELXS97* (Sheldrick, 2008 ▶); program(s) used to refine structure: *SHELXL97* (Sheldrick, 2008 ▶); molecular graphics: *SHELXTL* (Sheldrick, 2008 ▶); software used to prepare material for publication: *SHELXTL*.

## Supplementary Material

Crystal structure: contains datablocks I, global. DOI: 10.1107/S1600536810022361/im2208sup1.cif

Structure factors: contains datablocks I. DOI: 10.1107/S1600536810022361/im2208Isup2.hkl

Additional supplementary materials:  crystallographic information; 3D view; checkCIF report

## Figures and Tables

**Table 1:** Hydrogen-bond geometry (Å, °)

*D*—H⋯*A*	*D*—H	H⋯*A*	*D*⋯*A*	*D*—H⋯*A*
C7—H7*C*⋯O^i^	0.96	2.51	3.442 (4)	165
C1—H1*A*⋯O^ii^	0.93	2.60	3.414 (4)	145

Symmetry codes: (i) 
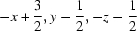
; (ii) 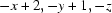.

**Fig. 1.**
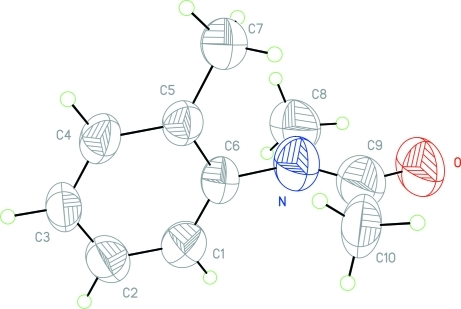
Molecular structure of (I) with the atom-numbering scheme. Displacement ellipsoids are drawn at the 50% probability level.

**Fig. 2.**
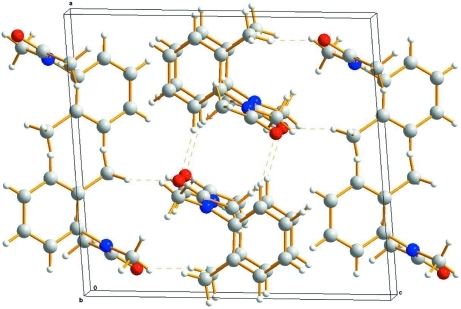
Packing diagram of (I). Hydrogen bonds are shown as dashed lines.

## supplementary crystallographic information

### Comment

The title compound, (I), contains acetyl group, which can react with different groups to prepare various function organic compounds. It is a kind of aromatic organic intermediate which can be used for many fields such as medicine. (Cai *et al.*, 2007). Herein we report its crystal structure.

In the molecule of (I), (Fig.1), the bond lengths (Allen *et al.*, 1987) and angles are within normal ranges. The N and C7 atoms are situated in the same plane as the benzene ring they are bonded to. The C—H···O intermolecular hydrogen bonds form a three dimensional network, which seems to be very effective in the stabilization of the crystal structure.

As can be seen from the packing diagram, (Fig. 2), the molecules are stacked along the *b* axis. There are also weak π-π interactions of benzene rings with a face-to-face stacking distance of 5.991 (4) Å.

### Experimental

The title compound, (I) was prepared by the literature method (Cai *et al.*, 2007). Crystals suitable for X-ray analysis were obtained by dissolving (I) (0.5 g) in ethyl acetate (20 ml) and evaporating the solvent slowly at room temperature for about 7 d.

### Refinement

All H atoms were positioned geometrically and constrained to ride on their parent atoms, with C—H = 0.93 Å and 0.96 Å for aromatic H and methyl group H, respectively. The *U*_iso_(H) = *xU*_eq_(C), where *x* = 1.2 for aromatic H, and *x* = 1.5 for other H.

### Crystal data

**Table:**

C_10_H_13_NO	*F*(000) = 352
*M**_r_* = 163.21	*D*_x_ = 1.142 Mg m^−^^3^
Monoclinic, *P*2_1_/*n*	Melting point: 328 K
Hall symbol: -P 2yn	Mo *K*α radiation, λ = 0.71073 Å
*a* = 11.288 (2) Å	Cell parameters from 25 reflections
*b* = 6.900 (1) Å	θ = 9–13°
*c* = 12.234 (2) Å	µ = 0.07 mm^−^^1^
β = 94.88 (3)°	*T* = 293 K
*V* = 949.5 (3) Å^3^	Block, colourless
*Z* = 4	0.30 × 0.20 × 0.10 mm

### Data collection

**Table:**

Enraf–Nonius CAD-4 diffractometer	1044 reflections with *I* > 2σ(*I*)
Radiation source: fine-focus sealed tube	*R*_int_ = 0.055
graphite	θ_max_ = 25.3°, θ_min_ = 2.4°
ω/2θ scans	*h* = 0→13
Absorption correction: ψ scan (North *et al.*, 1968)	*k* = −8→8
*T*_min_ = 0.978, *T*_max_ = 0.993	*l* = −14→14
3465 measured reflections	3 standard reflections every 200 reflections
1726 independent reflections	intensity decay: 1%

### Refinement

**Table:**

Refinement on *F*^2^	Primary atom site location: structure-invariant direct methods
Least-squares matrix: full	Secondary atom site location: difference Fourier map
*R*[*F*^2^ > 2σ(*F*^2^)] = 0.070	Hydrogen site location: inferred from neighbouring sites
*wR*(*F*^2^) = 0.180	H-atom parameters constrained
*S* = 1.00	*w* = 1/[σ^2^(*F*_o_^2^) + (0.060*P*)^2^ + 0.550*P*] where *P* = (*F*_o_^2^ + 2*F*_c_^2^)/3
1726 reflections	(Δ/σ)_max_ < 0.001
112 parameters	Δρ_max_ = 0.32 e Å^−^^3^
4 restraints	Δρ_min_ = −0.16 e Å^−^^3^

### Special details

**Table:**

Geometry. All e.s.d.'s (except the e.s.d. in the dihedral angle between two l.s. planes) are estimated using the full covariance matrix. The cell e.s.d.'s are taken into account individually in the estimation of e.s.d.'s in distances, angles and torsion angles; correlations between e.s.d.'s in cell parameters are only used when they are defined by crystal symmetry. An approximate (isotropic) treatment of cell e.s.d.'s is used for estimating e.s.d.'s involving l.s. planes.
Refinement. Refinement of *F*^2^ against ALL reflections. The weighted *R*-factor *wR* and goodness of fit *S* are based on *F*^2^, conventional *R*-factors *R* are based on *F*, with *F* set to zero for negative *F*^2^. The threshold expression of *F*^2^ > σ(*F*^2^) is used only for calculating *R*-factors(gt) *etc*. and is not relevant to the choice of reflections for refinement. *R*-factors based on *F*^2^ are statistically about twice as large as those based on *F*, and *R*- factors based on ALL data will be even larger.

### Fractional atomic coordinates and isotropic or equivalent isotropic displacement parameters (Å^2^)

**Table:**

	*x*	*y*	*z*	*U*_iso_*/*U*_eq_	
N	0.8267 (3)	0.3421 (5)	−0.0762 (2)	0.0917 (9)	
O	0.9121 (2)	0.5682 (4)	−0.1656 (2)	0.1110 (10)	
C1	0.8287 (3)	0.1854 (5)	0.1029 (3)	0.0744 (9)	
H1A	0.9111	0.1976	0.1095	0.089*	
C2	0.7737 (3)	0.0925 (4)	0.1832 (2)	0.0696 (8)	
H2A	0.8181	0.0387	0.2432	0.083*	
C3	0.6508 (3)	0.0794 (4)	0.1741 (2)	0.0660 (8)	
H3A	0.6121	0.0177	0.2285	0.079*	
C4	0.5867 (3)	0.1571 (4)	0.0853 (2)	0.0629 (8)	
H4A	0.5043	0.1465	0.0801	0.075*	
C5	0.6408 (2)	0.2526 (4)	0.0015 (2)	0.0554 (7)	
C6	0.7649 (3)	0.2603 (5)	0.0137 (2)	0.0682 (8)	
C7	0.5689 (3)	0.3319 (5)	−0.0977 (2)	0.0733 (9)	
H7A	0.5852	0.4676	−0.1048	0.110*	
H7B	0.4858	0.3138	−0.0895	0.110*	
H7C	0.5897	0.2648	−0.1621	0.110*	
C8	0.8631 (3)	0.1932 (7)	−0.1620 (3)	0.1025 (12)	
H8A	0.9102	0.2565	−0.2132	0.154*	
H8B	0.7930	0.1397	−0.2006	0.154*	
H8C	0.9087	0.0911	−0.1255	0.154*	
C9	0.8569 (3)	0.5129 (7)	−0.0876 (3)	0.0931 (10)	
C10	0.8173 (3)	0.6500 (5)	0.0004 (3)	0.0896 (10)	
H10A	0.8712	0.6398	0.0652	0.134*	
H10B	0.7386	0.6155	0.0177	0.134*	
H10C	0.8171	0.7808	−0.0264	0.134*	

### Atomic displacement parameters (Å^2^)

**Table:**

	*U*^11^	*U*^22^	*U*^33^	*U*^12^	*U*^13^	*U*^23^
N	0.0813 (19)	0.107 (2)	0.089 (2)	−0.0143 (17)	0.0181 (15)	0.0186 (16)
O	0.0849 (16)	0.150 (2)	0.1002 (17)	−0.0239 (16)	0.0217 (13)	0.0430 (16)
C1	0.0646 (18)	0.081 (2)	0.077 (2)	−0.0093 (16)	0.0022 (16)	0.0066 (18)
C2	0.090 (2)	0.0613 (17)	0.0576 (17)	−0.0028 (17)	0.0063 (15)	0.0046 (14)
C3	0.086 (2)	0.0624 (17)	0.0519 (15)	−0.0241 (16)	0.0178 (15)	0.0047 (14)
C4	0.0630 (17)	0.0684 (17)	0.0600 (17)	−0.0176 (14)	0.0214 (14)	−0.0081 (15)
C5	0.0601 (16)	0.0554 (15)	0.0517 (14)	−0.0082 (13)	0.0109 (12)	−0.0029 (12)
C6	0.0639 (18)	0.077 (2)	0.0650 (18)	−0.0171 (16)	0.0108 (14)	0.0132 (16)
C7	0.0699 (18)	0.083 (2)	0.0683 (18)	−0.0097 (17)	0.0101 (15)	0.0085 (17)
C8	0.083 (2)	0.159 (4)	0.070 (2)	−0.011 (2)	0.0350 (17)	0.007 (2)
C9	0.073 (2)	0.122 (3)	0.085 (2)	−0.017 (2)	0.0104 (16)	0.0221 (18)
C10	0.097 (2)	0.0670 (19)	0.106 (2)	−0.0223 (18)	0.0166 (19)	0.0247 (16)

### Geometric parameters (Å, °)

**Table:**

N—C9	1.238 (5)	C5—C6	1.396 (4)
N—C6	1.465 (4)	C5—C7	1.505 (4)
N—C8	1.549 (5)	C7—H7A	0.9600
O—C9	1.243 (4)	C7—H7B	0.9600
C1—C6	1.358 (4)	C7—H7C	0.9600
C1—C2	1.366 (4)	C8—H8A	0.9600
C1—H1A	0.9300	C8—H8B	0.9600
C2—C3	1.384 (4)	C8—H8C	0.9600
C2—H2A	0.9300	C9—C10	1.528 (5)
C3—C4	1.363 (4)	C10—H10A	0.9600
C3—H3A	0.9300	C10—H10B	0.9600
C4—C5	1.401 (3)	C10—H10C	0.9600
C4—H4A	0.9300		
			
C9—N—C6	127.2 (3)	C5—C7—H7A	109.5
C9—N—C8	117.6 (3)	C5—C7—H7B	109.5
C6—N—C8	115.1 (3)	H7A—C7—H7B	109.5
C6—C1—C2	120.9 (3)	C5—C7—H7C	109.5
C6—C1—H1A	119.5	H7A—C7—H7C	109.5
C2—C1—H1A	119.5	H7B—C7—H7C	109.5
C1—C2—C3	119.1 (3)	N—C8—H8A	109.5
C1—C2—H2A	120.4	N—C8—H8B	109.5
C3—C2—H2A	120.4	H8A—C8—H8B	109.5
C4—C3—C2	119.9 (3)	N—C8—H8C	109.5
C4—C3—H3A	120.0	H8A—C8—H8C	109.5
C2—C3—H3A	120.0	H8B—C8—H8C	109.5
C3—C4—C5	122.2 (3)	N—C9—O	122.6 (4)
C3—C4—H4A	118.9	N—C9—C10	114.2 (3)
C5—C4—H4A	118.9	O—C9—C10	123.1 (4)
C6—C5—C4	115.8 (3)	C9—C10—H10A	109.5
C6—C5—C7	122.6 (2)	C9—C10—H10B	109.5
C4—C5—C7	121.5 (2)	H10A—C10—H10B	109.5
C1—C6—C5	122.0 (3)	C9—C10—H10C	109.5
C1—C6—N	119.8 (3)	H10A—C10—H10C	109.5
C5—C6—N	118.1 (3)	H10B—C10—H10C	109.5
			
C6—C1—C2—C3	−1.7 (5)	C7—C5—C6—N	−2.5 (4)
C1—C2—C3—C4	0.6 (5)	C9—N—C6—C1	−91.7 (5)
C2—C3—C4—C5	−0.4 (4)	C8—N—C6—C1	84.8 (4)
C3—C4—C5—C6	1.1 (4)	C9—N—C6—C5	91.8 (4)
C3—C4—C5—C7	178.0 (3)	C8—N—C6—C5	−91.7 (4)
C2—C1—C6—C5	2.4 (5)	C6—N—C9—O	178.1 (3)
C2—C1—C6—N	−174.0 (3)	C8—N—C9—O	1.6 (6)
C4—C5—C6—C1	−2.1 (4)	C6—N—C9—C10	−3.6 (5)
C7—C5—C6—C1	−179.0 (3)	C8—N—C9—C10	179.9 (3)
C4—C5—C6—N	174.4 (3)		

### Hydrogen-bond geometry (Å, °)

**Table:**

*D*—H···*A*	*D*—H	H···*A*	*D*···*A*	*D*—H···*A*
C7—H7C···O^i^	0.96	2.51	3.442 (4)	165
C1—H1A···O^ii^	0.93	2.60	3.414 (4)	145

Symmetry codes: (i) −*x*+3/2, *y*−1/2, −*z*−1/2; (ii) −*x*+2, −*y*+1, −*z*.
